# Simulation of Shape Memory Alloy (SMA)-Bias Spring Actuation for Self-Shaping Architecture: Investigation of Parametric Sensitivity

**DOI:** 10.3390/ma13112485

**Published:** 2020-05-29

**Authors:** Hwang Yi

**Affiliations:** Architectural Design & Technology Lab, Department of Architecture, School of Engineering, Ajou University, Suwon 16499, Korea; hwy@ajou.ac.kr

**Keywords:** shape memory alloy, SMA, SMA actuator, sensitivity analysis, adaptive architecture

## Abstract

Parametric complexity of the thermomechanical shape memory alloy (SMA) model is one of the major barriers to advanced application of the SMA actuation in adaptive architecture. This article seeks to provide architectural practitioners with decision-making information about SMA actuator design parameters. Simulation-based global sensitivity analysis of an SMA-bias spring actuation model reveals that the SMA spring index (a spring’s outer diameter divided by its wire diameter) and stiffness of the bias spring are significant factors in both displacement and force exertion. Among all parameters, maximum output stroke and force largely depend on the temperature range at which the SMA spring operates. These findings also indicate a trade-off between the spring diameter and wire thickness, demonstrating that the output stroke and force tend to counter one another. Appropriate preloading and choice of an optimal spring index should be considered for desirable SMA motion.

## 1. Introduction

Shape memory alloy (SMA) as a smart material has been the subject of intensive research in various areas, including robotics, micromechanical systems, the aerospace and automotive industries, civil structural engineering, and biomedical sciences [1,2,3,4]. A wide range of industrial applications are also found in medical stents, unmanned aerial vehicles (UAVs), A/C vents, and so forth [1,5,6]. Compared to conventional electromagnetic motor actuators, SMAs offer noiseless operation, design flexibility, and resistance to functional degradation from dust or humidity with compact system configurations. In recent years, such unique technical benefits of SMAs in actuation have attracted considerable attention regarding their utility in building design disciplines; for instance, self-shaping kinetic architecture or climate-adaptive building with automatic environmental responsiveness [7,8,9,10].

SMAs are highly engineered materials and it is difficult for design professionals to gain a sufficient scientific understanding of the thermomechanical material behavior and underlying mechanisms of actuation. In building implementation, for example, it is of great concern to determine the exact lengths and positions of SMA parts in motion, thereby allowing for the largest possible stroke and force of an actuator to support the substantial scales and weights of building structures. However, many parameters related to sizing of SMA actuation are quite uncertain during the design stages of building projects, and a majority of existing SMA application approaches deal with SMA behaviors on smaller scales. Therefore, it is necessary to provide building designers with concise information related to parametric choice in large-scale SMA actuator design so that they are better and more efficiently informed of the thermomechanical SMA properties and the potential performance of SMA-driven kinetic building.

**Figure 1:**
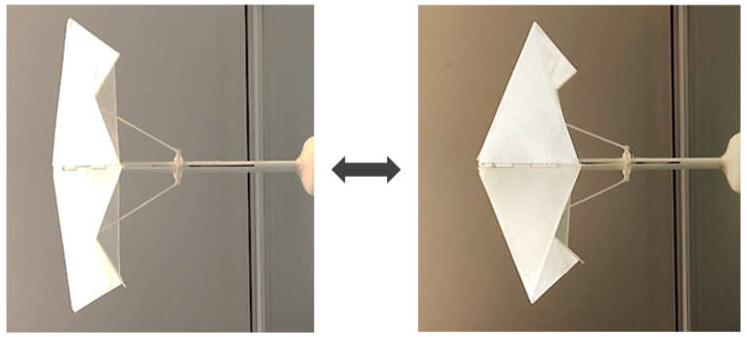
Example of SMA-bias extension spring actuation in architecture: 3D-printed parametric design of SMA-actuated shading [10].

SME has mostly been exploited to actuate the helical coil springs or tensile wires which conjoin other antagonistic (“bias”) mechanisms. An SMA-bias coupling mechanism has the capability to produce sizable actuation strains (four or more times greater extension/compression than their original lengths [11]), while the recovery strain potential of an SMA is limited to 6–8% [1]. Although complex types of bias mechanisms, such as multi-antagonistic or multi-input SMA actuators, have recently been developed [12,13], conventional SMA-bias coil-spring connections are preferred in architectural and building applications because they are easy to assemble and function robustly on a large scale (Figure 1).

Mechanical modeling can be used to predict deformation and force exertion by an SMA-bias spring actuation system. However, such systems are complicated and involve numerous parameters and constraints that are mutually concerned with the mechanical performance of the actuators [14]. Moreover, as the thermomechanical behavior of actuation is mainly characterized by its macroscopic aspects, several internal variables, e.g., the ratio of martensitic volume, cannot be measured without elaborate testing. Occasionally, a number of unknown parameters must be estimated before or during implementation [1,5]. Ambiguous parameter identification often results in increasing uncertainty in actuator design, which eventually propagates to a degradation in performance of an SMA-applied building.

**Figure 2:**
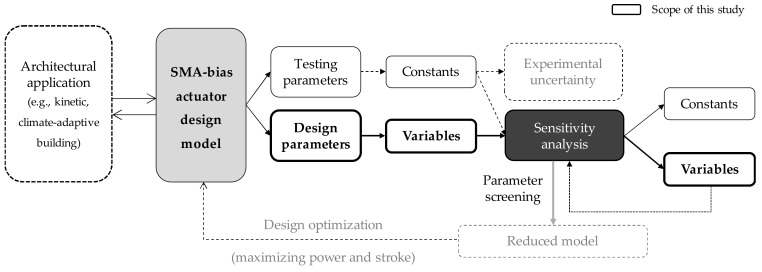
Scheme of the study scope and procedures.

A fundamental approach to mitigating parametric indeterminacy and improving the efficiency of decision-making is to conduct rigorous sensitivity analysis. By simulating a comprehensive SMA-bias system model, this study aims to quantitatively review and clarify the importance of the parameters through global sensitivity analysis (GSA). As shown in Figure 2, a thermomechanical design model of a general SMA-bias actuation system is presented and simulated for GSA. Focusing on design-related parameters, GSA is used to diagnose the sensitivity and interactive parametric influences of individual variables. For practical convenience, low-impact variables are classified as constants so that reduction in model complexity and parameter optimization can be further examined based on the findings of this study.

## 2. Materials and Methods

### 2.1. Theoretical SMA Constitutive Model

Derivation of a general prediction model for the prediction of shape-changing behavior is challenging because material parameters are identified with phenomenological investigation and experimental testing. Nonetheless, theoretic thermomechanical approaches have been well established by Tanaka [15], Brinson [16], and Liang and Rogers [17], and used to examine different types of SMA actuators [4]. Per unit mass of a local SMA body under static/quasi-static loading, the Clausius–Duhem inequality can be expressed in terms of specific internal energy as:

(1) $$\rho \left(\dot{u}-T\dot{s}\right)-\sigma :\nabla v\leq -\frac{q\cdot \nabla T}{T}$$

in which the left- and right-hand side represent the mechanical and thermal dissipation (entropy production rate), respectively. Using the Helmholtz free energy density, Φ≔u−Ts, and the elastic strain (ε) rate, $D≔\nabla v=\dot{\varepsilon }$, Equation (1) becomes:

(2) $$-\rho \left(\dot{\Phi }+\dot{T}s\right)+\sigma :D-\frac{q\cdot \nabla T}{T}\geq 0$$

and

(3) $$\sigma :\dot{\varepsilon }-\rho \dot{\Phi }-\frac{q\cdot \nabla T}{T}\geq 0$$

for isothermal processes, where ρ, σ, ∇v, u,q,T, ∇T, and s denote the mass density ($\rho =\underset{\Delta V\to 0}{\lim}\frac{\Delta m}{\Delta V}$, *m*: mass; *V*: volume), Cauchy stress tensor, velocity gradient, internal energy, heat flux tensor, temperature, temperature gradient, and entropy, respectively. The superposed dot notation refers to a time derivative, and $\nabla =\left(\frac{\partial }{\partial x},\frac{\partial }{\partial y},\frac{\partial }{\partial z}\right)$. It is a classical assumption to decouple mechanical and thermal contribution to dissipation, and we ignore the energy transformation into heat in the interest of the study. Furthermore, for SMAs, the functional dependency of Φ upon the internal state variables was proved by experiment [1] so that $\Phi :{\mathbb{R}}^{3}\to \mathbb{R}$, $\Phi \left(\varepsilon ,\;\xi ,\;T\right)$, where ξ denotes the martensitic volume fraction (MVF; 0≤ξ≤1). Therefore, Equation (3) can be rewritten as:

(4) $$\sigma :\dot{\varepsilon }-\rho \left(\frac{\partial \Phi }{\partial \varepsilon }:\dot{\varepsilon }+\frac{\partial \Phi }{\partial \xi }\dot{\xi }+\frac{\partial \Phi }{\partial T}\dot{T}\right)\geq 0$$

Equality is required for Equation (4) to hold for any arbitrary values. Hence,

(5) $$\sigma =\frac{\partial^{2}\psi }{\partial \varepsilon^{2}}:d\varepsilon +\frac{\partial^{2}\psi }{\partial \varepsilon \partial \xi }d\xi +\frac{\partial^{2}\psi }{\partial \varepsilon \partial T}dT$$

where *ψ* is the total Helmholtz free energy. The first term on the right-hand side accounts for the reversible elastic potential of SMA deformation. In this study, we consider linear loading evolution over discrete phase transition starting from the full austenite ($\varepsilon_{0}=0$**,** $\xi_{0}=0$). Then, Equation (5) can be expressed in a concise form such that:

(6) σ=C:ε+Ωξ+αΔT

with $\Omega \equiv \partial^{2}\psi /\partial \varepsilon \partial \xi$ and $\;\alpha \equiv \partial^{2}\psi /\partial \varepsilon \partial T$, where C, Ω, and α are the stiffness, transformation, and thermal expansion tensor, respectively. C is formulated by:

(7) $$C=C_{A}+\xi \left(C_{M}-C_{A}\right)$$

where the subscripts *A* and *M* denote the austenitic and martensitic state, respectively. Importantly, Brinson [16] suggests $\Omega ≔C:\varepsilon_{L}$, where $\varepsilon_{L}$ is maximum residual (recoverable) strain, which is typically about 4–6% and 8% at maximum in NiTi [1,5]. The thermal expansion coefficient is relatively quite small ($\sim 0.5\;–1.5\times 10^{-5}/K$) in most SMAs, and is therefore often neglected [1,18]. Therefore, Equation (6) becomes:

(8) $$\sigma ≅\left\{\begin{array}{c} {}[C_{A}+\xi \left(C_{M}-C_{A}\right)]:\left(\varepsilon +\varepsilon_{L}\xi \right),\;\;\varepsilon <0\\ {}[C_{A}+\xi \left(C_{M}-C_{A}\right)]:\left(\varepsilon -\varepsilon_{L}\xi \right),\;\;\varepsilon \geq 0 \end{array}\right.$$

The MVF reaches 100% in pure stress-induced martensite, whereas it becomes zero in full austenite. In other cases ($M_{f}<T<A_{f}$), SMAs always exist as a mixture of austenite and martensite, and ξ is approximated by the following formulas [2,19] with experimental curve-fitting parameters in an elementwise format

(9) $$\left\{\begin{array}{c} \xi_{M\to A}=\frac{\xi_{0}}{2}\left[\cos\left(a_{A}\left(T-A_{s}-\frac{\sigma_{ij}}{N_{A}}\right)\right)+1\right],\xi_{A\to M}=\frac{1-\xi_{0}}{2}cos\left(a_{M}\left(T-M_{f}-\frac{\sigma_{ij}}{N_{M}}\right)\right)+\frac{1+\xi_{0}}{2}\\ a_{A}=\frac{\pi }{A_{f}-A_{s}}\;,\;\;a_{M}=\frac{\pi }{M_{s}-M_{f}}\\ N_{A}\left(T-A_{f}\right)<\sigma_{ij,\;\;M\to A}<N_{A}\left(T-A_{s}\right),\;\;\;N_{M}\left(T-M_{s}\right)<\sigma_{ij,\;\;A\to M}<N_{M}\left(T-M_{f}\right) \end{array}\right.$$

where $\xi_{0}$ and $\sigma_{ij}$ are the initial MVF at the beginning of the current transformation and the stress tensor constituent, respectively. Note that the above are linear transformed expressions of a cosine function, $\xi =\cos\left(T\right)$, whose amplitude is determined by $\xi_{0}$. Equation (9) depends on the direction of the transition, i.e., martensite to austenite (M→A; heating) or vice versa, and *N_A_* and *N_M_* represent the influence of the loading and its direction to MVF. *N_A_* and *N_M_* can be experimentally computed from the slope of a σ−T curve [1] or derived from the Clausius–Claperyron relation [19], such as:

(10) $$\begin{array}{cc} N_{A}=\frac{\rho \Delta H_{A}}{T_{cr}\varepsilon_{L}}, & N_{M}=\frac{\rho \Delta H_{M}}{T_{cr}\varepsilon_{L}} \end{array}$$

where ΔH is the specific enthalpy (latent heat per unit mass) change during the phase transition, and the critical temperature *T_cr_* = (*A_s_* + *A_f_*)/2.

### 2.2. Modeling of 1-D SMA-Bias Spring Actuation

**Figure 3:**
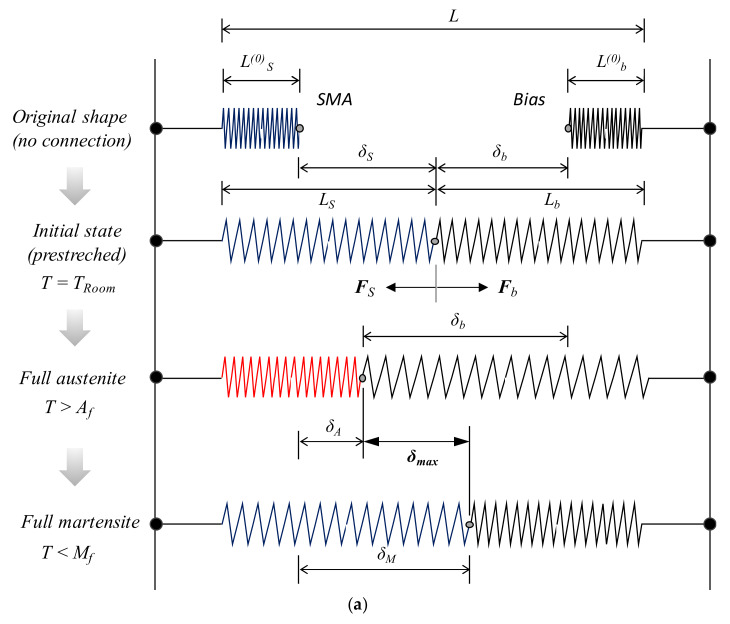

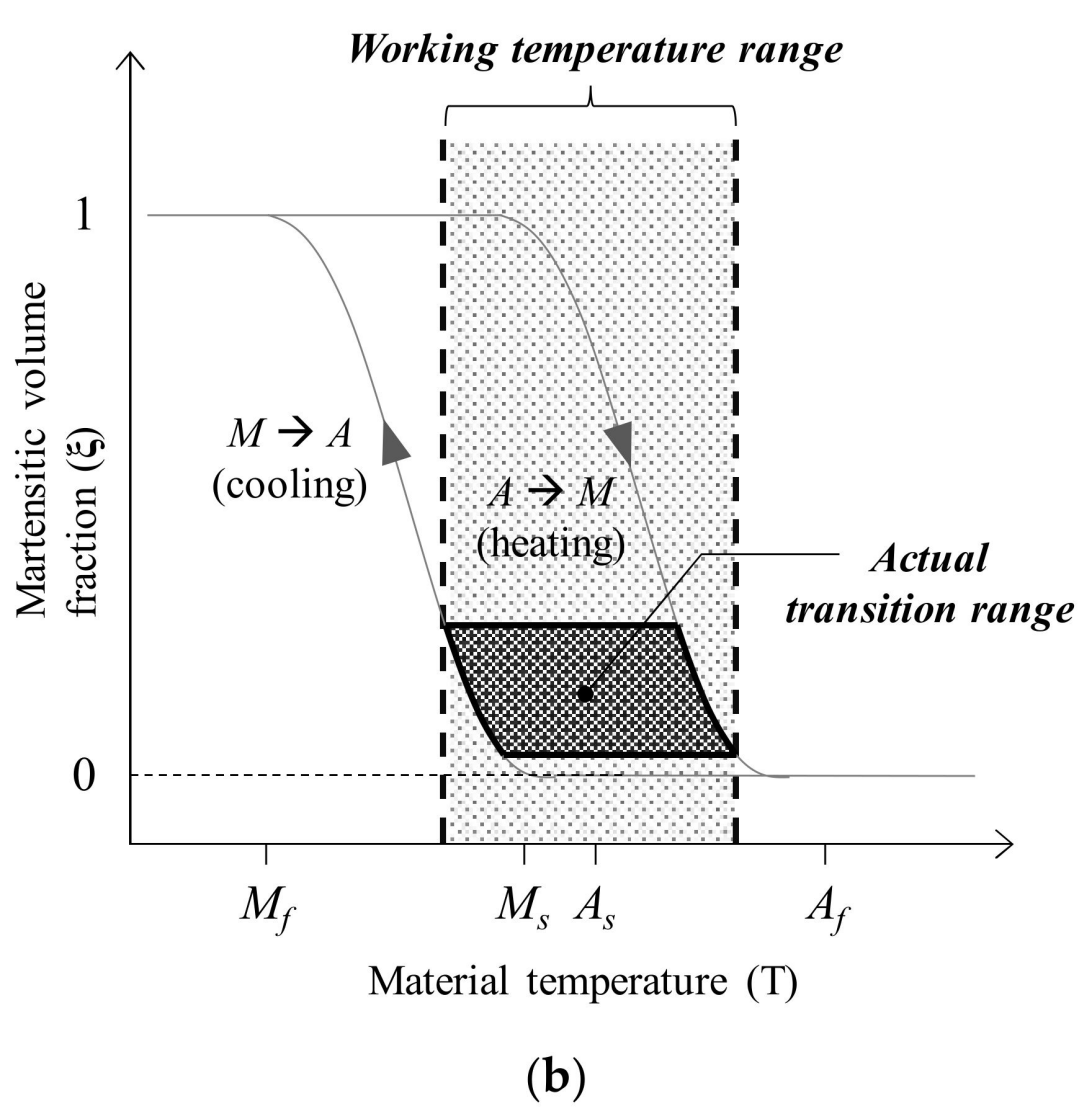
Schematic configuration of (**a**) SMA-bias actuation system and (**b**) MVF-temperature cycle (A: austenite, M: martensite).

**Figure 4:**
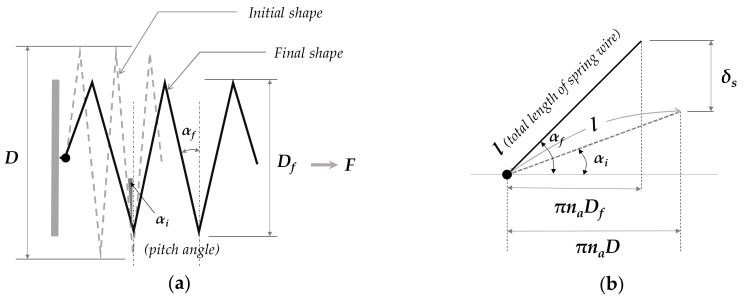
Parametric configuration of spring geometry: (**a**) Diameter change while in elongation; (**b**) Relationship between spring pitch angle and deflection [18].

Mechanical modeling of SMA-bias coil spring actuation has been intensively explored in various aspects [18,20,21]. Based on previous work, a concise expression of the modeling to estimate stroke and output force during the initial stage of actuator design was developed. Figure 3 represents an actuation scheme under investigation. Figure 3a depicts a simple actuation system with two springs (a helical SMA and a bias spring) held on two fixed sides, and Figure 3b illustrates MVF profiles given temperature changes. Coil springs under static axial loading produce shear stresses (τ), which consists of torsional ($\tau_{T}$) and direct shear ($\tau_{\mathrm{DS}}$) components. Hence, τ is given by:

(11) $$\tau =\tau_{T}+\tau_{\mathrm{DS}}=\left(1+\frac{1}{2C_{s}}\right)\frac{8FD}{\pi d^{3}}$$

where *F*, *D*, *d*, and $C_{s}$ denote axial force acting on the spring, original spring diameter, wire diameter, and spring index ($C_{s}=D/d$), respectively. Considering that martensite SMAs undergo a large degree of deflection, the amount of longitudinal extension (δ) should consider both the torque and bending effect, which results in:

(12) $$\delta =\frac{8n_{a}D_{f}^{3}}{d^{4}cos\alpha_{f}}\left(\frac{\cos^{2}\alpha_{f}}{G}+\frac{2\sin^{2}\alpha_{f}}{E}\right)F=\frac{8\left(L^{\left(0\right)}-d\right)D_{f}^{3}}{Gd^{5}}\left\{\frac{1+\nu \cos^{2}\alpha_{f}}{\left(1+\nu \right)cos\alpha_{f}}\right\}F$$

with $L^{\left(0\right)}=d\left(n_{a}+1\right)$ and $G=E/2\left(1+\nu \right)$. $n_{a}$, G, E, $L^{\left(0\right)}$, and ν are the number of active turns, shear modulus, Young’s modulus, initial spring length, and Poisson’s ratio (which is typically 0.33 in solids), respectively, and the subscripts *i* and *f* denote the initial and final geometric state, respectively. Note that the curvature effect (stress concentration) can be neglected if $C_{s}$ ≥ 4. In addition, given the geometry of spring extension (Figure 4), $D_{f}$ and δ can be represented as

(13) $$D_{f}=D\frac{\cos\alpha_{f}}{\cos\alpha_{i}}$$

(14) $$\delta =\frac{\pi n_{a}D}{cos\alpha_{i}}\left(\sin\alpha_{f}-\sin\alpha_{i}\right)=\frac{\pi \left(L^{\left(0\right)}-d\right)C_{s}}{cos\alpha_{i}}\left(\sin\alpha_{f}-\sin\alpha_{i}\right)$$

Therefore, Equation (12) becomes,

(15) $$\delta =\frac{8\left(L^{\left(0\right)}-d\right)C_{s}{}^{3}}{\left(1+\nu \right)Gd^{2}}\left\{\frac{\cos^{2}\alpha_{f}\left(1+\nu \cos^{2}\alpha_{f}\right)}{\cos^{3}\alpha_{i}}\right\}F$$

Since *τ* = *G*_*γ*_, where _*γ*_ is the shear strain, Equation (11) can be expressed as:

(16) $$F=\frac{G\pi d^{2}}{4\left(2C_{s}+1\right)}\gamma$$

For brevity of expression, let a new parameter Θ denote the trigonometric terms concerned with the pitch angles in Equation (15). Introducing Equation (16) to Equation (15), we obtain the following γ−δ relationship such as:

(17) $$\gamma =\frac{\left(1+\nu \right)\left(2C_{s}+1\right)}{2\pi C_{s}{}^{3}\left(L^{\left(0\right)}-d\right)\Theta }\delta$$

where $\Theta =\cos^{2}\alpha_{f}\left(1+\nu \cos^{2}\alpha_{f}\right)/\cos^{3}\alpha_{i}$. In Equations (6)–(8), we may substitute C, σ, and ε for G, τ, and γ, respectively, for one-dimensional tensile coil spring applications [18], such as $G=G_{A}+\xi \left(G_{M}-G_{A}\right)$. Note that the total shear strain of the SMA spring, $\gamma_{S}$, is the sum of the mechanical elastic ($\gamma_{e}$) and residual strain such that $\gamma_{S}=\gamma_{e}\pm \gamma_{L}\xi$, where $\gamma_{L}$ is the maximum residual shear strain and $\gamma_{L}:=\frac{\sqrt{3}}{2}\varepsilon_{L}$ by the Lagrangian equivalent definition ($\varepsilon_{L}=\sqrt{\varepsilon^{2}+4\gamma_{L}{}^{2}/3}$ and ε≈0). Representation of Equation (16) on behalf of the SMA spring force yields

(18) $$F_{S}=F_{e}+F_{R}=\frac{G_{S}d^{2}}{8}\left\{\frac{\left(1+\nu \right)\delta_{S}}{C_{s,\;S}{}^{3}(L_{S}^{\left(0\right)}-d)\Theta }+\omega \frac{\sqrt{3}\pi }{2C_{s,\;S}+1}\varepsilon_{L}\xi \right\},\;\;\omega =\left\{\begin{array}{c} 1,\;M\to A\\ -1,\;A\to M \end{array}\right.$$

where the subscripts *S*, *e*, and *R* stand for the SMA, elastic, and residual components, respectively. As depicted in Figure 3a, the equilibrium between $F_{S}$ and the opposing bias force ($F_{b}$) must be satisfied under static loading, which results in:

(19) $$k_{b}\delta_{b}=\frac{G_{S}d^{2}}{8}\left\{\frac{\left(1+\nu \right)\delta_{S}}{C_{s,\;S}{}^{3}(L_{S}^{\left(0\right)}-d)\Theta }+\omega \frac{\sqrt{3}\pi }{2C_{s,\;S}+1}\varepsilon_{L}\xi \right\}$$

(20) $$\delta_{S}+\delta_{b}=L-L_{S}^{\left(0\right)}-L_{b}^{\left(0\right)}$$

where $k_{b}$, $\delta_{b}$, and $L_{b}^{\left(0\right)}$ are the spring constant, displacement, and original length of the bias spring, respectively, and L denotes the effective total length of the SMA-bias actuator. In Equations (18)–(20), it is critical to constrain $\delta_{S}$ in such a way that

(21) $$0\;\leq \delta_{S}\leq L-L_{S}^{\left(0\right)}-L_{b}^{\left(0\right)}$$

and using Equation (17), equivalently,

(22) $$\frac{2\pi C_{s,\;S}{}^{3}\Theta \left(\gamma_{e}+0.5\sqrt{3}\varepsilon_{L}\xi \right)(L_{S}^{\left(0\right)}-d)}{\left(1+\nu \right)\left(2C_{s,\;S}+1\right)}\geq 0$$

(23) $$\frac{2\pi C_{s,\;S}{}^{3}\Theta \left(\gamma_{e}+0.5\sqrt{3}\varepsilon_{L}\xi \right)(L_{S}^{\left(0\right)}-d)}{\left(1+\nu \right)\left(2C_{s,\;S}+1\right)}\leq L-L_{S}^{\left(0\right)}-L_{b}^{\left(0\right)}$$

For compact expression, we assume that $2C_{s,\;S}+1=2C_{s,\;S}$ and $\gamma_{e}+\gamma_{L}=\gamma_{L}$ in Equation (23). Considering that $\gamma_{e}>\varepsilon_{L}\xi$, if M→A, and $\gamma_{e}\ll \varepsilon_{L}\xi$, if A→M in Equations (22) and (23), we obtain

(24) $$d\leq L_{S}^{\left(0\right)}\leq \frac{\left(1+\nu \right)\left(L-L_{b}^{\left(0\right)}\right)}{1+0.5\sqrt{3}\pi C_{s,\;S}{}^{2}\Theta \varepsilon_{L}\xi }$$

We find the lower bound of Equation (24) trivial, but the upper bound indicates that the SMA spring length is constrained by both the design of spring geometry (the spring constant and final pitch angle) and the material state (MVF). In Equation (24), it should be also emphasized that the shear modulus is not directly associated with the spring length limit. Now, introducing Equation (20) to Equation (19), and rearranging it for $\delta_{S}$, we obtain a comprehensive deformation function of SMA-bias coil spring actuation:

(25) $$\delta_{S}=\frac{C_{s,\;S}{}^{3}\Theta (L_{S}^{\left(0\right)}-d)\left\{8k_{b}\left(2C_{s,\;S}+1\right)\left(L-L_{S}^{\left(0\right)}-L_{b}^{\left(0\right)}\right)-\omega \sqrt{3}\pi G_{S}d^{2}\varepsilon_{L}\xi \right\}}{\left(2C_{s,\;S}+1\right)\{\left(1+\nu \right)G_{S}d^{2}+8k_{b}C_{s,\;S}{}^{3}\Theta (L_{S}^{\left(0\right)}-d)\}}$$

$\delta_{S}$ is assumed to be decomposed into the linear elastic and nonlinear residual terms such that:

(26) $$\delta_{S}=\delta_{e}+\delta_{R}\;\delta_{e}=\frac{L-L_{S}^{\left(0\right)}-L_{b}^{\left(0\right)}}{1+G_{S}d^{2}\left(1+\nu \right)/\lambda },\;\;\delta_{R}=\frac{-\omega \lambda \sqrt{3}\pi G_{S}d^{2}\varepsilon_{L}\xi }{8k_{b}\left(2C_{s,\;S}+1\right)\left(\left(1+\nu \right)G_{S}d^{2}+\lambda \right)}$$

with $\lambda =8k_{b}C_{s}{}^{3}\Theta \left(L_{S}^{\left(0\right)}-d\right)$. Replacing $\sigma_{ij}$ with shear stress in Equation (9), ξ is represented as:

(27) $$\xi =\left\{\begin{array}{c} \frac{\xi_{0}}{2}\left[\cos\left(a_{A}\left(T-A_{s}-\frac{\sigma_{S}}{N_{A}}\right)\right)+1\right],\;\;M\to A\\ \frac{1-\xi_{0}}{2}cos\left(a_{M}\left(T-M_{f}-\frac{\sigma_{S}}{N_{M}}\right)\right)+\frac{1+\xi_{0}}{2},\;\;A\to M \end{array}\right.$$

in which $\sigma_{S}$ accounts for the axial stress-induced MVF due to mechanical elongation ($\delta_{e}$). We may assume that $\sigma_{S}$ is zero, because the elastic strain (*ε*) is negligible in the axially elongated section of the coil spring.

### 2.3. Sensitivity Analysis (SA) and Monte Carlo Approach to Simulation

Sensitivity analysis is used for quantitative parameter screening and significance identification. Among several methods (OAT, WALS, FAST, etc.), Sobol’s variance-based technique is employed in this study, because it enables us to factor in any type of nonlinear variables and high-order interactive effects in a global domain [22,23]. The Sobol analysis results in a direct metric of sensitivity that is obtained via decomposition of overall variance with respect to input. Given the function of a model *f* and a random input vector ***X*** such as

(28) $$f:X\to Y,\;\;X=\{X_{i}|X_{i}\in \left[0,1\right],\;i=1,2,\ldots ,\;n\},$$

the total output variance, $V\left(Y\right)$, can be decomposed as

(29) $$V\left(Y\right)=\overset{n}{\underset{s=1}{\sum }}\overset{n}{\underset{i_{1}<\ldots <i_{s}}{\sum }}V_{i_{1},\ldots ,i_{s}}=\overset{n}{\underset{i=1}{\sum }}V_{i}+\overset{n-1}{\underset{i=1}{\sum }}\overset{n}{\underset{j=i+1}{\sum }}V_{i,j}+V_{1,2,\ldots ,n},\;\;1\leq i_{1}\leq \ldots \leq i_{s}\leq n$$

and the Sobol indices are defined as the contribution of each conditional input variance to the total variance of $f\left(X\right)$

(30) $$S_{i_{1},\ldots ,i_{n}}=\frac{V_{X_{i_{1},\ldots ,i_{n}}}\left(E_{X_{\sim i_{1},\ldots ,i_{n}}}\left(Y|X_{i_{1},\ldots ,i_{n}}\right)\right)}{V\left(Y\right)}=\frac{V_{i_{1},\ldots ,i_{n}}}{V\left(Y\right)}$$

where the subscripts $i_{s}$ and *i*,*j,…,n* are a generic expression of conditional and multi-dimensional combination of variables, respectively. Note that it is assumed that each input variable is independent and distributed uniformly in a unit hypercube space. The total-effect Sobol index (*ST*) considering all high-order interactive effects of a variable $X_{i}$ is calculated as

(31) $$ST_{i}=1-\frac{V_{X_{\sim i}}\left(E_{X_{i}}\left(Y|X_{\sim i}\right)\right)}{V\left(Y\right)},\;\;\overset{n}{\underset{i=1}{\sum }}ST_{i}\geq 1$$

In the Sobol framework, the Monte Carlo simulation (MCS) approach is used for the evaluation of a full range of parameter variation in high-dimensional space. The MCS is a stochastic computational algorithm that searches randomly generated sets of variable samples. Since no assumption is made between input and output, the MCS is numerically intuitive and any type of probabilistic/non-probabilistic data can be put into the MCS procedure. Having determined the parameters to be varied, the uncertainty of the model can be drawn by propagating parameter variation through the MCS. The Latin hypercube method is employed to reduce the domain size of variable space and increase sampling efficiency for the MCS in this experiment.

### 2.4. Study Parameters

The SMA modeling parameters in 2.2 are largely divided into design and testing parameters according to the purpose of this study. Design parameters primarily concern designers’ decision-making on the spring details and the sizing of an actuator, while testing parameters are identified by instrumental measurement. Each parameter contributes to the performance of SMA-bias spring actuation, and the association of individual parameters and related dependent variables are shown in Table 1. For SA, a numerical range of each parameter is defined with a lower and upper bound, so it is randomly generated to run the models with the sets of parameters through MCS. Before SA, we may assume that the design parameters are basically variables and the testing parameters are constants which need further uncertainty investigation. In the Sobol method, when the *ST* value of a variable is no greater than 0.05, we may consider it as a constant. Note that this method cannot capture the cause(s) of the input variability or the source(s) of the contribution. The numerical finding is followed by the interpretation of parametric impacts.

## 3. Results and Discussion

### 3.1. Experimental Parameter Investigation

**Figure 5:**
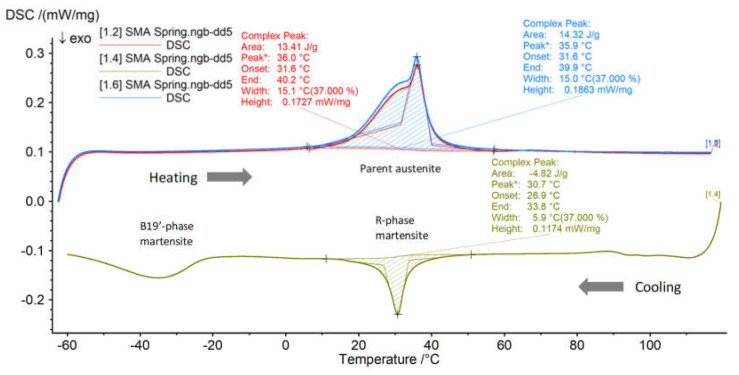
DSC curves: The peak during cooling indicates a symmetric R-phase transformation (blue: austenite upon the first heating; red: austenite during the second heating).

To determine the testing parameter values, a sample NiTi coil spring (Ni-50.9 *wt*.%) with a *C*_s,*S*_ of 7.5 was manufactured (SME Ltd., Hwaseong, Gyeonggi, Korea), assuming *L* = 300 and $L_{S}^{\left(0\right)}$ ≤ 150, and its mechanical properties were measured. The values listed in Table 2 are referenced for the model simulation. Differential scanning calorimetry (DSC) results (Figure 5) reveal that the sample material exhibits an *M_f_* and *A_f_* of 26.9 °C and 40.1 °C, with an Δ*H*_*M*_ and Δ*H*_*A*_ of 4.82 and 13.87, respectively (DSC apparatus: NETZSCH DSC 200 F3 Maia). Among the testing parameters, Δ*H*_*M*_, Δ*H*_*A*_ and *ρ* are assumed to be constant in all simulations and Sas.

**Figure 6:**
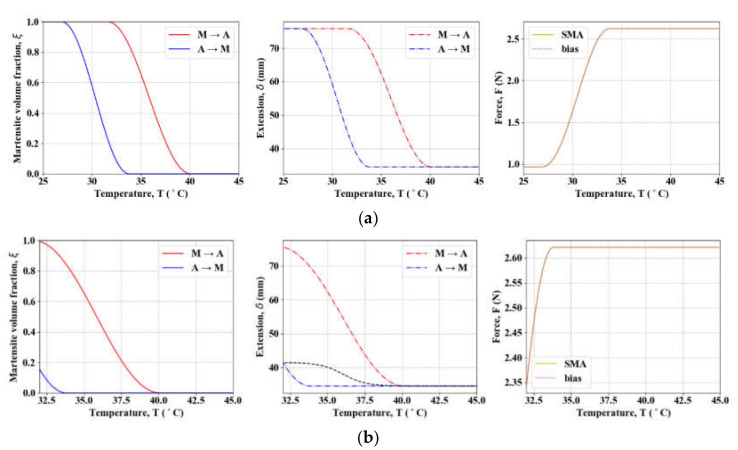

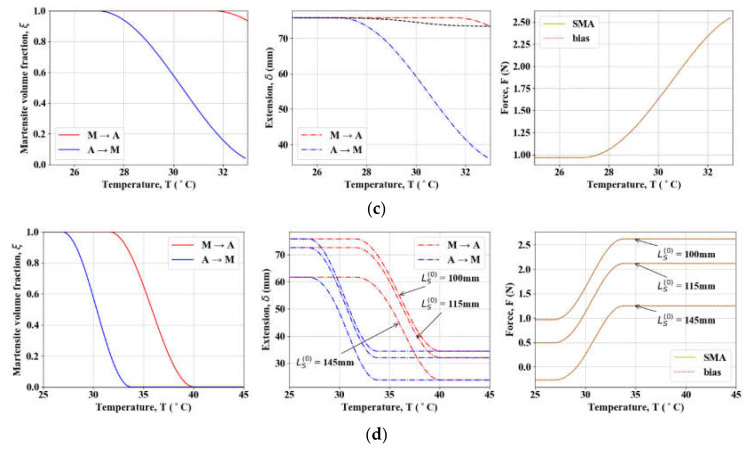
Simulation of SMA phase transformation behavior (*L* = 300, $L_{S}^{\left(0\right)}$ = 100, $L_{b}^{\left(0\right)}$ = 100): (**a**) full cycle; (**b**) incomplete cooling; (**c**) incomplete heating; (**d**) behavior of multiple length SMA springs (black dotted lines in (b) and (c) indicate logistic approximation of recovery *M* → *A*.

On the other hand, MVF is considered nearly zero in full austenite [24]. The SMA coil is simulated by setting $\xi_{0}$ = 0 for the forward cycle (A→M) and $\xi_{0}$ = 1 for the reverse (M→A). In Figure 6, notice that incomplete thermal cycles (in cases in which terminal temperatures of an actuation do not reach either *M_f_* or *A_f_*) result in much smaller SMA extension than its full potential (Figure 6a–c). Figure 6d demonstrates that the actuation stroke becomes greater as $L_{S}^{\left(0\right)}$ lengthens. However, the magnitude of the available force may tend, on the contrary, to decrease.

### 3.2. SA: Spring Pitch Angle Variation

**Figure 7:**
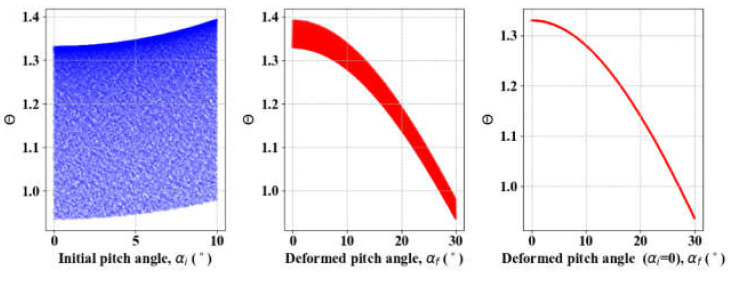
SA result of spring pitches and Θ: $S_{\alpha_{i}}$ = 0.018, $S_{\alpha_{f}}$ = 0.982.

Equation (14) indicates that a larger spring index (*C*_s_) and pitch angle (*α*) are advantageous to obtaining greater extension. However, in industrial practice, a *C*_s_ of 4–16 is recommended and 6–12 is preferred by manufacturers so that *α*_*i*_ and *α*_*f*_ are less than 5°–10° and 20°–30°, respectively, to ensure robust contraction [25]. Figure 7 shows the variation in Θ with *α*_*i*_ of [0°, 10°] and *α*_*f*_ of [0°, 30°] with 10,000 samples generated using SALib. SA results in *S*_*α*_*i*__ = 0.018 and *S*_*α*_*f*__ = 0.982, which indicates that *α*_*i*_ is far less important than *α*_*f*_. Θ is almost constant with an *α*_*i*_ less than 4°, and we obtain Θ ∈ [0.94, 1.33] with mean (*µ*) of 1.1 and standard deviation (*σ*) of 0.12 on *α*_*i*_ ∈ [0°, 4°].

### 3.3. SA: Limit of Initial SMA Length ($L_{S,max}^{\left(0\right)}$)

**Figure 8:**
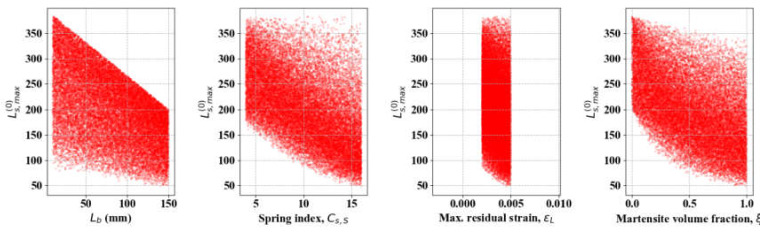
SA results of $L_{S,\;\max}^{\left(0\right)}$-related variables (*L* = 300).

From the results in Section 3.2, we may consider Θ as a constant by taking its mean hereinafter (Θ = 1.1); then, Equation (25) is simulated with $L_{b}^{\left(0\right)}$ ∈ [5, 150], *C*_*s*,*S*_ ∈ [4, 16], ε_*L*_ ∈ [0.002, 0.005], and ξ ∈ [0, 1]. Note that ε_*L*_ does not exceed 1% in R-phase transformation, and generally ranges from 0.2% to 0.5% [26]. The recommended practical limit is 4% only if stress-applied B19 martensite is considered [1,27]. The SA results in Table 3 and Figure 8 show that *ξ* and *C*_*s*, *S*_ are the most constraining variables in the determination of $L_{S,max}^{\left(0\right)}$. The results show that $L_{b}^{\left(0\right)}$ is slightly less important and ε_*L*_ is negligible because *ST*_ε*L*_ < 0.05 (Table 3).

### 3.4. SA: SMA Displacement ($\delta_{S}$)

**Figure 9:**
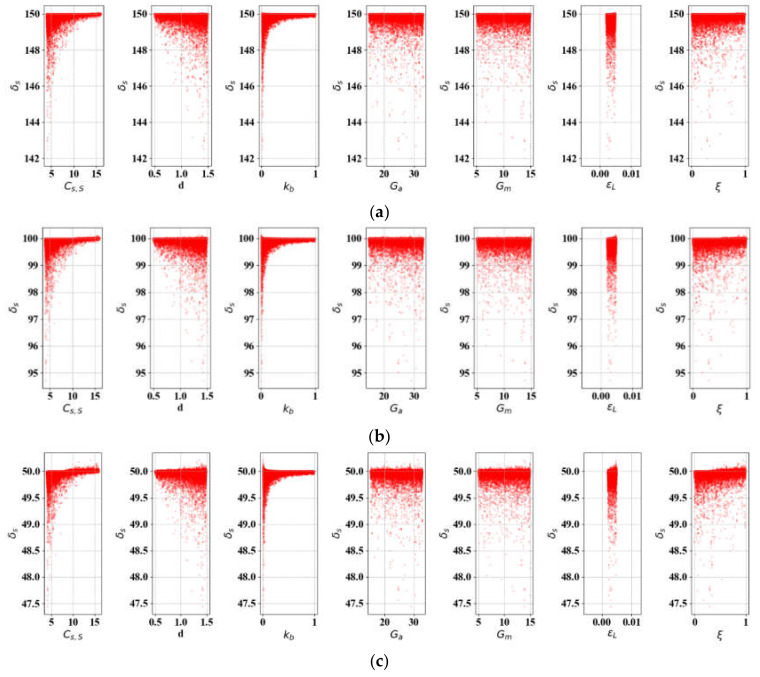
SA of $\delta_{S}$-related variables (*L* = 300, $L_{S}^{\left(0\right)}$ = 100): (**a**) $L_{b}^{\left(0\right)}$ = 50; (**b**) $L_{b}^{\left(0\right)}$ = 100; (**c**) $L_{b}^{\left(0\right)}$ = 150.

**Figure 10:**
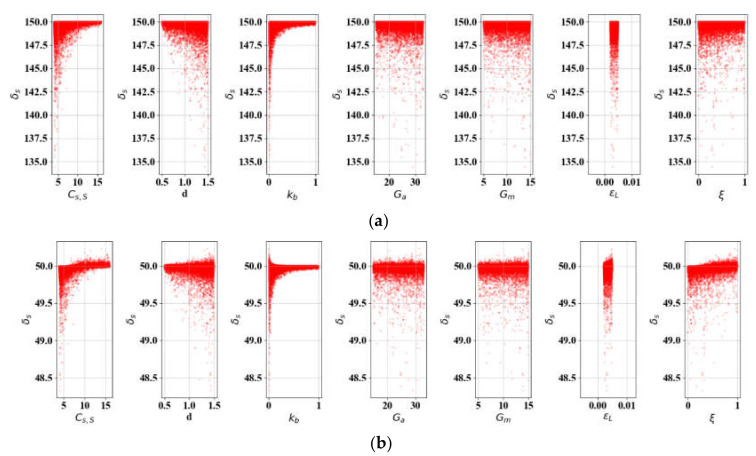
SA of $\delta_{S}$-related variables (*L* = 300, $L_{b}^{\left(0\right)}$ = 100): (**a**) $L_{s}^{\left(0\right)}$ = 50; (**b**) $L_{s}^{\left(0\right)}$ = 150.

**Figure 11:**
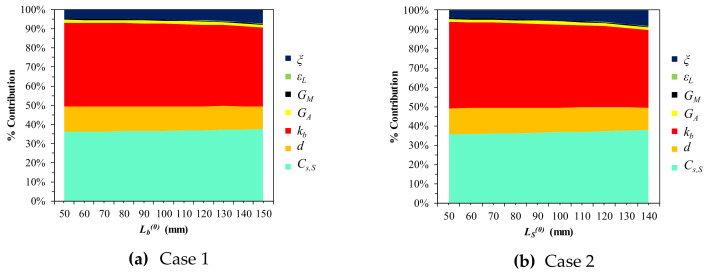
Parametric percentage contribution to SMA spring elongation ($\delta_{S}$): (**a**) Variation in bias spring length (L = 300, $L_{S}^{\left(0\right)}$ = 100); (**b**) Variation in initial SMA spring length (L = 300, $L_{b}^{\left(0\right)}$ = 100).

For $\delta_{S}$, it was identified through pre-examination of SA that the initial spring lengths ($L_{S}^{\left(0\right)}$ and $L_{b}^{\left(0\right)}$) are enormously influential (responsible for more than 90% variations in $\delta_{S}$) in hiding the contributions of other variables. Therefore, $L_{S}^{\left(0\right)}$ and $L_{b}^{\left(0\right)}$ are set as discretely varying constants in this experiment. We set $L_{S}^{\left(0\right)}$ and *L* to 50 and 300, respectively, and Equation (25) was run on $\varepsilon_{L}$ ∈ [0.002, 0.005], *d* ∈ [0.5, 1.5], $k_{b}$ ∈ [0.01, 0.2], *G_A_* ∈ [19.84, 32.72], and *G_M_* ∈ [5.79, 16.13] by referring to the literature [1,2,3,4,10,19]. Figure 9 shows the results of $\delta_{S}$ per parameter combinations of 10,000 samples, increasing $L_{b}^{\left(0\right)}$ by 50 mm (Case 1: $L_{S}^{\left(0\right)}$ fixed), and Figure 10 presents variation in $\delta_{S}$ when $L_{S}^{\left(0\right)}$ increases from 50 to 150 mm (Case 2: $L_{b}^{\left(0\right)}$ fixed). The SA results over $L_{S}^{\left(0\right)}$, $L_{b}^{\left(0\right)}$∈ [50, 150] indicate that $C_{s,\;S}$ and $k_{b}$ are the most significant variables for SMA elongation in both cases, followed by *d* and ξ. In both cases (Table 4 and Figure 11), it is noteworthy that ξ is increasingly important as $L_{b}^{\left(0\right)}$ and $L_{S}^{\left(0\right)}$ lengthen, while the contribution of $k_{b}$ slightly diminishes. We expect that the impact of ξ could be ignored if the SMA were shorter. These results confirm that the variations in *G_A_*, *G_M_*, and $\varepsilon_{L}$ exhibit very little contribution to $\delta_{S}$, and they can be considered constants in SMA elongation.

### 3.5. SA: Actuation Force ($F_{S}$)

**Figure 12:**
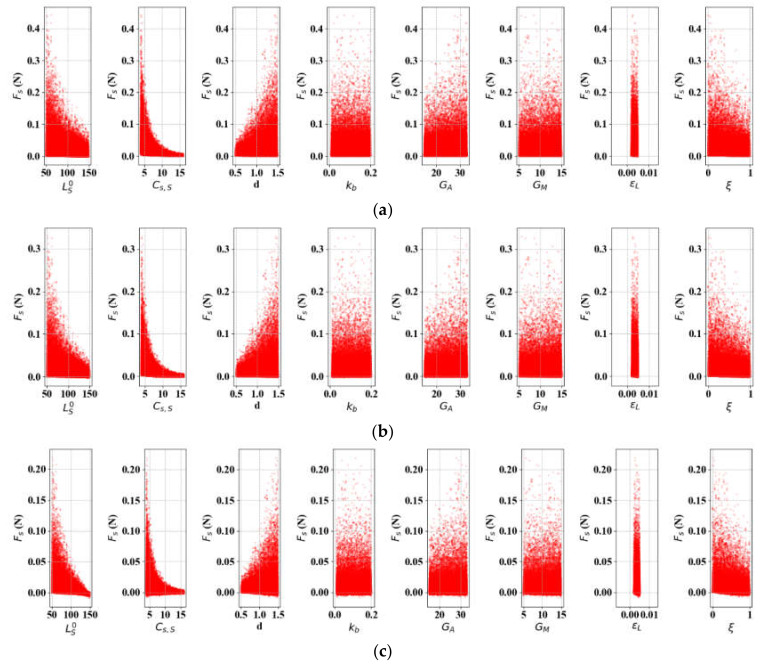
SA results of $F_{S}$-related variables (L = 300): **(a)** $L_{b}^{\left(0\right)}$ = 50; **(b)** $L_{b}^{\left(0\right)}$ = 100; **(c)** $L_{b}^{\left(0\right)}$ = 150.

**Figure 13:**
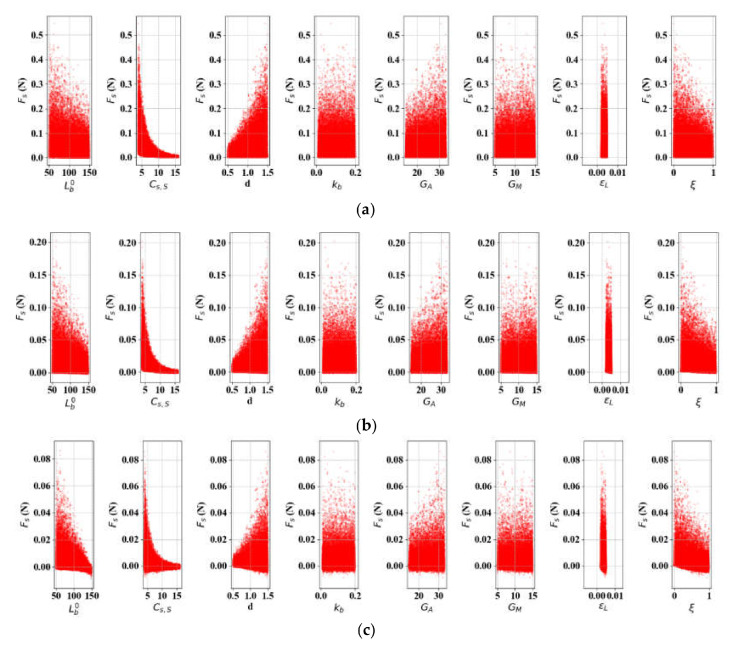
SA results of $F_{S}$- related variables (*L* = 300): (**a**) $L_{b}^{\left(0\right)}$ = 50; (**b**) $L_{b}^{\left(0\right)}$ = 100; (**c**) $L_{b}^{\left(0\right)}$ = 150.

**Figure 14:**
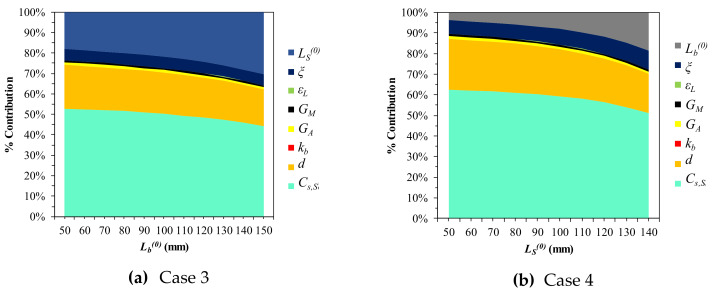
Parametric percentage contribution to the SMA force ($F_{S}$): (**a**) Variation in bias spring length; (**b**) Variation in initial SMA spring length.

The actuation force equation (18) was simulated for SA with the same parameters as in $\delta_{S}$. In the same manner as Section 3.4, two cases were examined over $F_{S}$. Figure 12 shows Case 3, in which $L_{b}^{\left(0\right)}$ changes by 10 mm in discrete steps, and, in Figure 13 (Case 4), $L_{S}^{\left(0\right)}$ varies in the same manner. Note that $L_{S}^{\left(0\right)}$ and $L_{b}^{\left(0\right)}$ are treated as continuous variables where appropriate, since the influence of the initial lengths in $F_{S}$ is not as critical as it is in $\delta_{S}$. The total Sobol index values in Table 5 reveal that $C_{s,\;S}$ is the most sensitive factor in both cases. It is important to note that $k_{b}$ is negligible, unlike in the cases on displacement. In Figure 14, we find that the impact of $C_{s,\;S}$ and *d* decrease, while the spring length parameters ($L_{S}^{\left(0\right)}$ and $L_{b}^{\left(0\right)}$) become more important. $L_{S}^{\left(0\right)}$ is more sensitive if $L_{b}^{\left(0\right)}$ is determined. In Case 4, the internal state factor (ξ) is slightly more sensitive than it is in Case 3. In all cases, *G_A_*, *G_M_*, and $\varepsilon_{L}$ are only slightly influential, and $\varepsilon_{L}$ in particular can be ignored.

### 3.6. SA: Maximum Output Stroke and Force

As illustrated in Figure 3b, the SMA spring in the presence of bias is generally expected to actuate in temperatures ranging between *M_f_* and *A_f_*. Thus, the cyclic deflection and force change between the martensitic and austenitic states, and the maximum output stroke ($\Delta \delta_{S}$) and force ($\Delta F_{S}$) characterize the final performance of the actuation. Assuming that the actuation takes place between a certain high (*T_H_* ≤ *A_f_*) and low temperature (*T_L_* ≤ *M_f_*), $\Delta \delta_{S}$ and $\Delta F_{S}$ can be expressed as:

(32) $$\Delta \delta_{S}=\delta_{S,T_{L}}-\delta_{S,\;T_{H}}\;$$

(33) $$\Delta F_{S}=F_{S,T_{H}}-F_{S,T_{L}}$$

**Figure 15:**
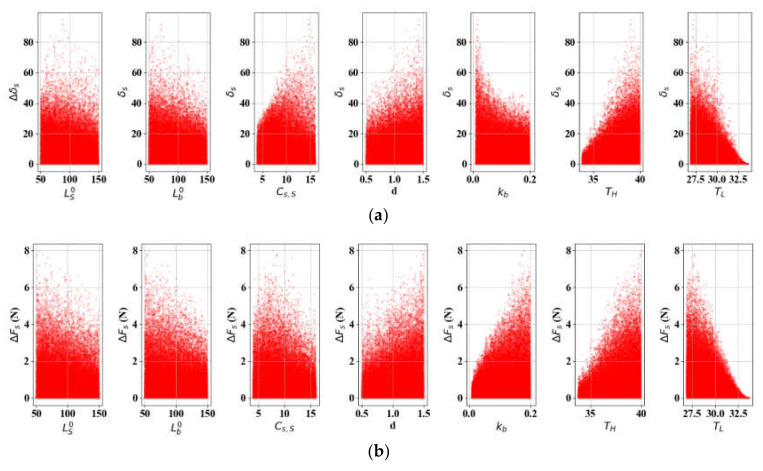
SA results (*L* = 300): (**a**) $\Delta \delta_{S}$; (**b**) $\Delta F_{S}$.

**Figure 16:**
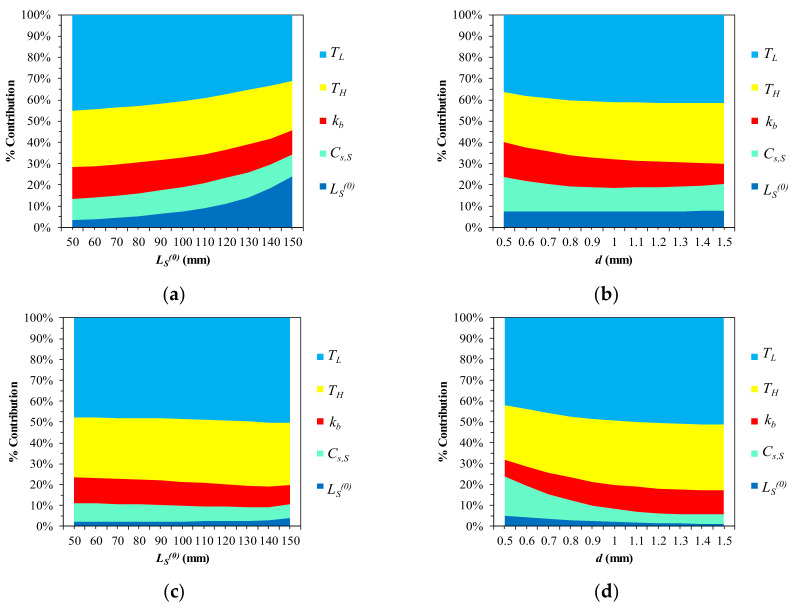
Trends in parametric contribution (L = 300): (**a**) $\Delta \delta_{S}$ (*d* = 0.9); (**b**) $\Delta \delta_{S}$ ($L_{b}^{\left(0\right)}$ = 100); (**c**) $\Delta F_{S}$ (*d* = 0.9); (**d**) $\Delta F_{S}$ ($L_{b}^{\left(0\right)}$ = 100).

Since we find that *ST*s of *G_A_*, *G_M_*, and $\varepsilon_{L}$ are not very significant, in almost all instances of $\delta_{S}$ and $F_{S}$ (Table 4 and Table 5), they are set as constants ($\varepsilon_{L}$ = 0.0035). Refer to the values in Table 2. $\Delta \delta_{S}$ and $\Delta F_{S}$ are simulated using Equations (25) and (18) on $L_{S}^{\left(0\right)}$, $L_{b}^{\left(0\right)}\in$ [50, 150], $C_{s,\;S}$ ∈ [4, 16], *d* ∈ [0.5, 1.5], $k_{b}$ ∈ [0.01, 0.2], *T_H_* ∈ [33, 40], and *T_L_* ∈ [26, 33] with a temperature change interval of 0.01 °C. Figure 15 and Table 6 suggest that the terminal operation temperatures (*T_H_* and *T_L_*) are highly impactful for both $\Delta \delta_{S}$ and $\Delta F_{S}$ (especially *T_L_*), whereas $L_{b}^{\left(0\right)}$ is much less important. Furthermore, the wire diameter (*d*) is not very sensitive, in contrast to Cases 1 through 4. $k_{b}$ and $C_{s,\;S}$ are the third and fourth major parameters, which are almost equally sensitive. Figure 16 displays the percentage contributions and trends in the parameters by reintroducing $L_{b}^{\left(0\right)}$ and *d* as discrete variables. Figure 16a shows that the initial SMA length gains importance gradually, reducing *T_L_*. The increasing sensitivity of *T_L_* and *T_H_* leads to the overall contribution change in Figure 16b. The sharp decrease in the contribution of $C_{s,\;S}$ along with the thickening of the wire is noticeable in Figure 16d. $k_{b}$ gradually decreases as the wire thickens and the bias spring lengthens. In all cases, the contribution of $L_{S}^{\left(0\right)}$ is less than 10%.

## 4. Discussion and Concluding Remarks

### 4.1. Rankings of Parameter Importance in SMA-Bias Actuator Design

Examining SMA behavior through simulation is an effective way to determine parameters and constraints during design phases. Table 7 lists the sensitivity ranking of inputs in numbered order. Large index springs are advantageous to the output stroke if the actuator length is limited [28], implying that small spring diameters are likely prone to deflection, although *D* is concealed in the parameter list. On the other hand, note that the spring coefficient of the bias spring is critical to SMA elongation. This result suggests that the optimal choice of a bias spring with a proper dimension should be emphasized to obtain desirable actuator performance. The importance of the SMA spring index and the bias spring coefficient can also be found in $\Delta \delta_{S}$ and $\Delta F_{S}$ (Table 7). In most cases, the maximum residual strain is insignificant, since it is too small in most SMAs undergoing R-phase transformation. However, MVF should be considered important in the estimation of an SMA elongation limit as well as $F_{S}$, if the length of an SMA spring is predefined.

### 4.2. Trade-Off between Output Stroke and Force

The major parametric behavior of $\delta_{S}$ and $F_{S}$ (Section 3.4 and Section 3.5) reflects the inherent trade-off between actuation stroke and force. A comparison of Figure 10 and Figure 12 indicates that the spring index, wire diameter, and MVF show opposite tendencies in $\delta_{S}$ and $F_{S}$, i.e., the greater the maximum stroke, the smaller the output force. This finding suggests that an accurate estimate of the martensite property (ξ) as well as optimization of the critical parameter values ($C_{s,\;S}$ and $k_{b}$) is required to predict the actual performance of SMA-bias spring actuators.

### 4.3. Temperature Dependency of Actuator Performance

It is evident that MVF, a function of material temperatures, is crucial to the mechanical behavior of SMAs (Figure 6), as it is directly involved with the elastic modulus. By the same token, actuator output performance tends to depend mainly on the bound of the terminal operating temperatures, which is similar to the findings of a previous study using the Ivshin–Pence model [29]. To obtain the desired output stroke and force, Figure 6 and Section 3.6 suggest that SMA ingredients be properly manufactured for the R-phase *M_s_* to be no greater than *A_s_* so that elongation and recovery are clearly separated in the morphing of the internal SMA structures. Increasing the gradient of the temperature-MVF curve can benefit sharp austenitic/martensitic transition with short temporal delay in the kinematic regime change.

Indeed, the thermomechanical explanation of SMA behavior is complicated. An actual maximum stroke is contingent upon the internal material state and various uncertain environmental factors, therefore presumably being less than the maximum estimate from any model. The theoretical maximum and minimum of ξ (1 and 0) is achievable under extremely constrained conditions ($T\ll M_{f}$ or $T\gg A_{f}$), which may not be feasible with air-cooling/heating SMAs in construction applications.

### 4.4. Reduction in Model Complexity and Uncertainty in SA

Given the SA results, the complexity of the actuation model can be avoided by eliminating insignificant parameters, along with dimension reduction. In particular, the small contribution of the residual strain may reduce the nonlinearity of the SMA transformation, increasing its dependency on the shear modulus change primarily induced by temperature. That said, the study findings should not be exaggerated. The mechanical properties of SMAs are phenomenological, and it is not likely that we could define a single deterministic model applicable to all types. Accordingly, the appropriate setting of likely parameter values is important to assure that they are suitably sampled for SA within the interest of any given investigation. Should specific upper/lower bounds of a parameter domain be unavailable, then one may approximate them by referring to the literature. However, this may increase output uncertainty and, as such, risk being ungeneralizable to SA outcomes. Moreover, the Sobol SA quantifies only input variances on model output, and none of the results explain an absolute level of parametric contribution. Thus, further study is required to quantify the propagation of parametric SA uncertainty with probabilistic definitions of the parameter values.

## Figures and Tables

**Table 1:** Experimental parameter categorization.

Model Output	Design Parameter (■)								Testing Parameter (□)							
	L	$L_{S}^{\left(0\right)}$	$L_{b}^{\left(0\right)}$	$C_{s,\;S}$	d	$k_{b}$	$\alpha_{i}$	$\alpha_{f}$	$G_{A}$	$G_{M}$	$\varepsilon_{L}$	ξ	$M_{f}$	$M_{s}$	$A_{s}$	$A_{f}$
Θ ^1^							■	■								
$L_{S,max}^{\left(0\right)}$ ^1^	■		■	■			■	■			□	□	□	□	□	□
$\delta_{S}$ ^1^	■	■	■	■	■	■	■	■	□	□	□	□	□	□	□	□
$\delta_{e}$	■	■	■	■	■	■	■	■	□	□						
$\delta_{R}$		■		■	■	■	■	■	□	□	□	□	□	□	□	□
$F_{S}$ ^1^		■		■	■		■	■	□	□	□	□	□	□	□	□
γ		■		■	■		■	■	□	□	□	□	□	□	□	□
τ		■		■	■		■	■	□	□	□	□	□	□	□	□
$\Delta \delta_{S}$ ^1^	■	■	■	■	■	■	■	■	□	□	□	□	□	□	□	□
$\Delta F_{S}$ ^1^		■		■	■		■	■	□	□	□	□	□	□	□	□

^1^ Target output variable under investigation.

**Table 2:** Mechanical properties of the test material.

ρ (kg/m^3^)	*D* (mm)	*d* (mm)	$C_{s,\;S}$	*G_A_* (GPa)	*G_M_* (GPa)	$\Delta H_{A}\;(J/g)\;$	$\Delta H_{M}\;(J/g)$
6.45E+3	6.75	0.9	7.5	31.35	15.24	13.87	4.82
***M_f_*** (°C)	***M_s_*** (°C)	***A_s_*** (°C)	***A_f_*** (°C)	***T_cr_*** (°C)	***ν***	***α_i_*** (°)	***ε_L_***
26.9	33.8	31.6	40.1	35.9	0.33	0	0.0035

**Table 3:** Total Sobol indices (*ST*) of $L_{S,max}^{\left(0\right)}$ parametrs.

$ST_{L_{b}^{\left(0\right)}}$	$ST_{C_{s,\;S}}$	$ST_{\varepsilon_{L}}$	$ST_{\xi }$
0.317 (0.0161^1^)	0.347 (0.021^1^)	0.043 (0.0041^1^)	0.355 (0.0231^1^)

^1^ Confidence interval.

**Table 4:** Total Sobol indices (*ST*) of $\delta_{S}$ (*L* = 300): Case 1 (left) and Case 2 (right).

$L_{b}^{{\left(0\right)}_{1}}$	$ST_{C_{s,\;S}}$		$ST_{d}$		$ST_{k_{b}}$		$ST_{G_{A}}$		$ST_{G_{M}}$		$ST_{\varepsilon_{L}}$		$ST_{\xi }$		$L_{S}^{{\left(0\right)}_{2}}$
50	0.644	*0.627*	0.231	*0.233*	0.779	*0.784*	0.027	*0.026*	0.011	*0.012*	0	*0.000*	0.083	*0.070*	*50*
60	0.646	*0.632*	0.231	*0.233*	0.778	*0.782*	0.027	*0.026*	0.011	*0.012*	0.001	*0.000*	0.084	*0.074*	*60*
70	0.648	*0.637*	0.230	*0.232*	0.777	*0.781*	0.027	*0.026*	0.011	*0.011*	0.001	*0.000*	0.086	*0.078*	*70*
80	0.651	*0.643*	0.230	*0.231*	0.776	*0.779*	0.027	*0.026*	0.011	*0.011*	0.001	*0.000*	0.088	*0.082*	*80*
90	0.654	*0.650*	0.229	*0.230*	0.775	*0.776*	0.027	*0.027*	0.010	*0.011*	0.001	*0.001*	0.091	*0.087*	*90*
100	0.657	*0.657*	0.228	*0.228*	0.773	*0.773*	0.027	*0.027*	0.010	*0.010*	0.001	*0.001*	0.094	*0.094*	*100*
110	0.662	*0.667*	0.227	*0.226*	0.771	*0.769*	0.027	*0.027*	0.010	*0.010*	0.001	*0.002*	0.098	*0.102*	*110*
120	0.667	*0.678*	0.226	*0.223*	0.768	*0.764*	0.027	*0.027*	0.010	*0.009*	0.002	*0.002*	0.103	*0.114*	*120*
130	0.674	*0.691*	0.224	*0.219*	0.765	*0.757*	0.027	*0.028*	0.009	*0.008*	0.002	*0.004*	0.110	*0.130*	*130*
140	0.683	*0.707*	0.221	*0.214*	0.761	*0.749*	0.027	*0.028*	0.009	*0.007*	0.003	*0.007*	0.120	*0.153*	*140*
150	0.695	*0.725*	0.218	*0.208*	0.754	*0.737*	0.027	*0.028*	0.008	*0.007*	0.005	*0.012*	0.136	*0.191*	*150*

^1^$L_{s}^{\left(0\right)}$ = 100, ^1^ $L_{b}^{\left(0\right)}$ = 100.

**Table 5:** Total Sobol indices (*ST*) of *F*_*S*_ (×1E-1, *L* = 300): Case 3 (left) and Case 4 (right).

$L_{b}^{\left(0\right)}$	$ST_{L_{b}}$	$ST_{C_{s,\;S}}$		$ST_{d}\;$		$ST_{k_{b}}$		$ST_{G_{A}}$		$ST_{G_{M}}$		$ST_{\varepsilon_{L}}\;$		$ST_{\xi }\;$		$ST_{L_{S}}$	$L_{S}^{\left(0\right)}$
50	*0.50*	8.09	*8.43*	3.27	*3.34*	0.00	*0.00*	0.19	*0.19*	0.15	*0.14*	0.00	*0.00*	0.85	*0.89*	0.87	*50*
60	*0.58*	8.08	*8.41*	3.26	*3.31*	0.00	*0.00*	0.19	*0.19*	0.15	*0.14*	0.00	*0.00*	0.85	*0.92*	0.88	*60*
70	*0.67*	8.06	*8.39*	3.25	*3.28*	0.00	*0.00*	0.19	*0.19*	0.15	*0.13*	0.00	*0.00*	0.86	*0.94*	0.89	*70*
80	*0.78*	8.03	*8.36*	3.24	*3.25*	0.00	*0.00*	0.19	*0.18*	0.15	*0.13*	0.00	*0.00*	0.86	*0.98*	0.90	*80*
90	*0.93*	8.00	*8.32*	3.23	*3.22*	0.00	*0.00*	0.19	*0.18*	0.15	*0.13*	0.00	*0.01*	0.87	*1.01*	0.92	*90*
100	*1.12*	7.97	*8.27*	3.22	*3.18*	0.00	*0.00*	0.19	*0.18*	0.15	*0.13*	0.00	*0.01*	0.88	*1.06*	0.93	*100*
110	*1.37*	7.93	*8.19*	3.20	*3.13*	0.00	*0.00*	0.19	*0.18*	0.15	*0.13*	0.01	*0.01*	0.88	*1.11*	0.95	*110*
120	*1.71*	7.88	*8.09*	3.18	*3.07*	0.00	*0.00*	0.19	*0.18*	0.15	*0.12*	0.01	*0.02*	0.89	*1.18*	0.97	*120*
130	*2.17*	7.82	*7.95*	3.16	*2.99*	0.00	*0.00*	0.18	*0.18*	0.15	*0.12*	0.01	*0.03*	0.90	*1.26*	1.00	*130*
140	*2.81*	7.74	*7.73*	3.13	*2.89*	0.00	*0.00*	0.18	*0.17*	0.15	*0.11*	0.01	*0.04*	0.91	*1.36*	1.02	*140*
150	*3.70*	7.64	*7.42*	3.10	*2.78*	0.00	*0.00*	0.18	*0.16*	0.14	*0.11*	0.01	*0.06*	0.92	*1.47*	1.05	*150*

**Table 6:** Total Sobol indices (*ST*) of $\Delta \delta_{S}$ and $\Delta F_{S}$ parameters.

	$ST_{L_{S}^{\left(0\right)}}$	$ST_{L_{b}^{\left(0\right)}}$	$ST_{C_{s,\;S}}$	$ST_{d}$	$ST_{k_{b}}$	$ST_{T_{H}}$	$ST_{T_{L}}$
$\Delta \delta_{S}$	0.107	0.074	0.157	0.099	0.16	0.406	0.573
$\Delta F_{S}$	0.101	0.06	0.155	0.119	0.193	0.364	0.549

**Table 7:** Evaluation of parametric impact with contribution ranking.

Output	$L_{S}^{\left(0\right)}$	$L_{b}^{\left(0\right)}$	$C_{s,\;S}$	d	$k_{b}$	$G_{A}$	$G_{M}$	$\varepsilon_{L}$	ξ	$T_{H}\;$	$T_{L}$
$L_{S,max}^{\left(0\right)}$		3	2					*	1		
$\delta_{S}$ ^1^	-	-	2	3	1	*	*	*	4		
$\delta_{S}$ ^2^	-	-	2	3	1	*	*	*	4		
$F_{S}$ ^3^	2	-	1	3	*	*	*	*	4		
$F_{S}$ ^4^	-	3	1	2	*	*	*	*	4		
$\Delta \delta_{S}$	5	7	4	6	3	-	-	-		2	1
$\Delta F_{S}$	6	7	4	5	3	-	-	-		2	1

^1^ Case 1, ^2^ Case 2, ^3^ Case 3, ^4^ Case 4, -: set as a constant, *: insignificant.

## Nomenclature

**Abbreviation**	Description	Unit
Φ	Specific Helmholtz free energy	J/g
*A*, *M*	Austenite, martensite	-
$C_{A}$, $C_{M}$	Austenitic (*A*) and martensitic (*M*) alloy stiffness	N/mm^2^
ξ	Martensite volume fraction	-
$N_{A}$, $N_{M}$	Stress-temperature curve gradient in austenite and martensite	-
ΔH	Specific enthalpy (latent heat)	J/g
Θ	Coefficient of spring pitch angles	-
*L*	Total length of actuator spring connection	mm
$L_{b}^{\left(0\right)}$	Original length of bias spring	mm
$L_{S}^{\left(0\right)}$	Original length of shape memory alloy (SMA) spring	mm
$G_{S}$	SMA spring shear modulus	N/mm^2^
$\delta_{S}$	SMA spring deflection	mm
$\Delta \delta_{S}$	Output stroke of actuation	mm
$F_{S}$	SMA spring force	N
$\Delta F_{S}$	Output force of actuation	N
$C_{s,\;S}$	SMA spring index	-
$k_{b}$	Bias spring coefficient	N/mm
$\varepsilon_{L}$	Residual strain	-
*ST*	Total Sobol index	-
$T_{cr}$	Critical temperature	°C
